# Early Pregnancy Body Mass Index and Experiences of Gendered Racial Microaggressions in a Multiracial, Multiethnic Prospective Cohort

**DOI:** 10.1111/birt.70051

**Published:** 2026-01-14

**Authors:** Kimberly B. Glazer, Natalie Boychuk, Frances M. Howell, Micki Burdick, Sarah Nowlin, Sheela Maru, Oluwadamilola Oshewa, Maria Monterroso, Erynne Jackson, Katharine McCarthy, Alva Rodriguez, Jennifer Lewey, Elizabeth A. Howell, Lisa Levine, Teresa Janevic

**Affiliations:** 1 Department of Obstetrics and Gynecology, University of Pennsylvania Perelman School of Medicine, Philadelphia, Pennsylvania, USA; 2 Department of Biostatistics, Epidemiology and Informatics, University of Pennsylvania Perelman School of Medicine, Philadelphia, Pennsylvania, USA; 3 Department of Population Health Science and Policy, Icahn School of Medicine at Mount Sinai, New York City, New York, USA; 4 Department of Obstetrics, Gynecology and Reproductive Science, Icahn School of Medicine at Mount Sinai, New York City, New York, USA; 5 Department of Epidemiology, Columbia University Mailman School of Public Health, New York City, New York, USA; 6 Department of Women and Gender Studies, University of Delaware, Newark, Delaware, USA; 7 Center for Nursing Research and Innovation, Mount Sinai Health System, New York City, New York, USA; 8 Department of Global Health and Health Systems Design, Icahn School of Medicine at Mount Sinai, New York City, New York, USA; 9 Elmhurst Department of Obstetrics & Gynecology, NYC Health + Hospitals, New York City, New York, USA; 10 Division of Cardiovascular Medicine, University of Pennsylvania Perelman School of Medicine, Philadelphia, Pennsylvania, USA

**Keywords:** discrimination, gender, microaggression, obesity, obstetrics, race, weight

## Abstract

**Objective:**

Weight bias is a source of stigma in healthcare, and obesity is disproportionately prevalent among Black and Hispanic individuals of reproductive age. However, relationships between body size and other forms of discrimination in perinatal settings remain poorly understood. Our objective was to examine the association between body mass index (BMI) and gendered racial microaggressions (GRM)—everyday discriminatory experiences related to race and gender—during perinatal care.

**Methods:**

We studied a prospective cohort of Asian, Black, and Hispanic (“Global Majority”) individuals who gave birth in four New York City and Philadelphia hospitals from March 2022–March 2023. Early pregnancy BMI was ascertained from weight and height recorded at first prenatal visit. Participants completed the validated GRM Scale, adapted for perinatal context by a community working group, during the birth hospitalization. We examined mean ± standard deviation (SD) GRM Scale score by BMI class and measured associations between BMI and GRM using multivariable Tweedie regression.

**Results:**

Of 368 participants, 27.2% had normal weight (18.5 kg/m^2^ ≤ BMI < 25), 29.9% overweight (25 ≤ BMI < 30), 32.6% class I-II obesity (30 ≤ BMI < 40), and 10.3% class III obesity (BMI ≥ 40). Thirty-seven percent of participants reported experiencing at least one instance of GRM during perinatal care. Mean ± SD GRM Scale score (higher = more frequent) increased with BMI class, from 1.7 ± 3.8 among those with normal weight to 4.8 ± 9.3 with class III obesity; associations persisted after adjusting for age, education, parity, and late prenatal care.

**Conclusion:**

BMI is associated with perinatal GRM among Global Majority individuals. Intersectional research on weight bias and discrimination, incorporating patient and provider perspectives, is warranted for inclusive, respectful perinatal care.

## 1 ∣ Introduction

Obesity is the most common health condition among individuals of reproductive age and is disproportionately prevalent among Black and Hispanic pregnant and birthing people. Approximately 39% of non-Hispanic (NH) Black, 32% of Hispanic, 26% of NH White, and 10% of NH Asian US females have pre-pregnancy body mass index (BMI) ≥ 30 kg/m^2^ [1].

Gendered racial microaggressions (GRM) represent a form of discrimination characterized by “subtle and everyday verbal, behavioral, and environmental expressions of oppression based on the intersection of one’s race and gender” [2]. Such discrimination can adversely affect patient experience, engagement in care, and health trajectories. In the context of perinatal health care, racial-ethnic discrimination is associated with reduced postpartum visit completion [3] and care continuity among Black women with cardiometabolic conditions [4]. Moreover, biases related to race and gender may overlap with other stigmatized identities such as having a larger body size. Intersectionality is a theoretical and analytic framework that acknowledges that no person has an essential single social identity, but rather multiple identities that are experienced simultaneously and reflect interlocking systems of privilege and oppression [5]. Previous research indicates that negative attitudes toward and treatment of individuals with higher weight are common in healthcare settings [6, 7]. However, the role of body size in perinatal experiences among Global Majority (i.e., African, Asian, Latin American, or Indigenous descent) birthing people is understudied, warranting research on BMI and GRM in perinatal care.

Using prospective data from a multiracial, multiethnic pregnancy cohort, our objective was to evaluate associations between early pregnancy BMI and patients’ experiences of GRM in prenatal and childbirth care.

## 2 ∣ Materials and Methods

### 2.1 ∣ Study Participants

The prospective coronaVirus Impact on Birth Equity (VIBE) Study enrolled 419 Asian, Black, and Hispanic (“Global Majority”) patients from March 2022 to March 2023 during their childbirth hospitalization in four Philadelphia and New York City hospitals [8]. Individuals were eligible if they identified as Asian, Black, or Hispanic, spoke English or Spanish, and had a working cell phone to use a text-based platform for research activities. All participants provided informed consent, and the protocol was approved by an institutional review boards at the Perelman School of Medicine at the University of Pennsylvania, the Icahn School of Medicine at Mount Sinai, and the Columbia University Irving Medical Center and received approval from the NYC Health and Hospitals Research Committee. We excluded individuals with missing exposure data (Gendered Racial Microaggressions Scale; *n* = 20) or early pregnancy BMI (*n* = 28), and underweight participants (*n* = 3) from our analyses for a final sample of *n* = 368 participants.

### 2.2 ∣ Gendered Racial Microaggressions (GRM)

We assessed GRM using a modified nine-item version of Lewis and Neville’s GRM Scale, adapted by the VIBE study team for use with Global Majority pregnant and birthing people in perinatal settings (Figure S1) [9]. The Lewis and Neville GRM Scale was designed to assess microaggressions experienced by Black women [2], including 23 items that examine the frequency of experienced GRM scored on a 6-point Likert scale for frequency (*0 = never to 5 = once a week or more*) [2]. Development of the adapted scale has been reported elsewhere [9]. In brief, all adaptations were made in collaboration with the study’s Community Working Group (CWG), comprised of racially and ethnically diverse community health workers, obstetricians, doulas, educators, and reproductive justice advocates with lived experience and expertise working with Global Majority birthing people in New York City and Philadelphia. The CWG provided input on capturing experiences of GRM during perinatal care and to ensure that the adapted questions resonated with a diverse group of birthing people. The adapted version includes nine items, prompted by the question stem: “At any time during your pregnancy care or in the hospital to deliver your baby, did any of these things happen to you?” Items include: “I have felt unheard,” “My comments have been ignored,” “Someone challenged my autonomy,” “Someone has tried to ‘put me in my place’,” “I have been disrespected,” “I felt excluded from services or resources,” “Someone assumed I did not have much to contribute to the conversation regarding my care,” “Someone told me to calm down,” “Someone accused me of being angry when speaking assertively” (Table S1). The respondent’s perspective is inferred from their reported race and gender positionality. Psychometric analysis of the adapted GRM Scale showed good construct and criterion validity in hospital-based perinatal care settings among multiracial and multiethnic patient populations and in both English and Spanish [9].

### 2.3 ∣ Weight Measures

We ascertained early pregnancy BMI (weight in kilograms/height in meters squared) using the weight and height measured at the first prenatal visit in the electronic medical record (EMR) [10]. We categorized early pregnancy BMI (first prenatal weight in kilograms/height in meters^2^) per Institute of Medicine (IOM) guidelines (normal weight: 18.5 kg/m^2^ ≤ BMI < 25 kg/m^2^; overweight: 25 kg/m^2^ ≤ BMI < 30 kg/m^2^; class I-II obesity: 30 kg/m^2^ ≤ BMI < 40 kg/m^2^, and class III obesity: BMI ≥ 40 kg/m^2^).

### 2.4 ∣ Covariates

We examined characteristics of our study population including maternal age (continuous), education (less than high school, high school complete, at least some college), parity (nulliparous vs. multiparous), gestational age at birth (continuous), late prenatal care initiation (≥ 2nd trimester), and a composite indicator for hypertensive disorders in pregnancy (any diagnosis of chronic hypertension, gestational hypertension, or preeclampsia). We ascertained maternal age, parity, gestational age at birth, prenatal care initiation, and hypertensive disorders from the EMR. Chronic hypertension, gestational hypertension, and preeclampsia were identified if documented in the medical chart according to American College of Obstetricians and Gynecologists guidelines described previously [8]. Self-reported race-ethnicity and education were collected from the baseline survey administered during the birth hospitalization by trained research coordinators. We examined gestational weight gain, defined as the difference between weight closest to the birth hospitalization and the first prenatal visit weight recorded in the EMR. We measured gestational weight gain as a *z*-score based on previously published BMI class-specific weight-gain-for-gestational-age charts [11], which measure the number of standard deviations an individual’s gestational weight gain is from the mean weight gain among all individuals who delivered at a given gestational age.

### 2.5 ∣ Statistical Analyses

We assessed the distribution of sample characteristics by BMI class using Chi-square and two-sample *t*-tests. We examined mean GRM Scale score by early pregnancy BMI class and used Tweedie regression with a log link to evaluate the association between early pregnancy BMI and continuous GRM Scale score. Tweedie regression accommodates zero-inflated, right-skewed outcome data [12]. We observed a skewed distribution of GRM Scale scores in our sample, as shown in the supplemental material (Figure S1). When used with a log link, Tweedie regression provides an estimate of the multiplicative effect of BMI class on the expected value of GRM Scale score. We adjusted multivariable Tweedie regression models for confounders defined a priori using Directed Acyclic Graphs (Figure S2), including maternal age, education, parity, and late prenatal care entry. We conducted sensitivity analyses additionally adjusting for race-ethnicity and explored effect measure modification in models stratified by race-ethnicity (categorized as Asian; Black; Hispanic, including White Hispanic; and other, including multiple races and not specified). In addition to overall GRM Scale score, we examined the percent of participants in each BMI class who endorsed individual GRM Scale items.

### 2.6 ∣ Sensitivity Analysis for Timing of First Prenatal Weight

The first recorded prenatal visit date in the EMR was after the first trimester (≥ 14 weeks of gestation) for 34.1% (*n* = 89) of participants and, as a result, early pregnancy weight was ascertained in the second trimester for these individuals. To increase the likelihood that our exposure measure of early pregnancy BMI preceded the experience of GRM, we conducted a sensitivity analysis setting early pregnancy weight to missing and imputing early pregnancy weight values for participants with a first prenatal visit ≥ 14 weeks of gestation [13]. We conducted multiple imputation assuming data were missing at random and using a fully conditional specification approach [14]. We included all covariates listed previously as well as the weight measured closest to the birth hospitalization in the imputation model; each of these variables was missing for < 1% of participants. Categorical variables were imputed using the discriminant function method and continuous variables with predictive mean matching. We generated one hundred imputed data sets using PROC MI, repeated the regression analyses of associations between BMI and GRM on each of the imputed data sets using PROC GENMOD, and pooled parameter estimates and standard errors across the one hundred analyses using PROC MIANALYZE. All analyses were conducted using SAS, Version 9.4 (SAS Institute).

## 3 ∣ Results

The mean ± standard deviation (SD) age of the 368 participants was 30.2 ± 5.9 years (Table 1). Forty-one percent (41.3%) identified as Hispanic, 37.8% as non-Hispanic Black, 10.6% as non-Hispanic Asian, and 10.3% as other or mixed race-ethnicity. Early pregnancy BMI classification identified 27.2% of participants as people with normal weight, 29.9% with overweight, 32.6% with class I–II obesity, and 10.3% with class III obesity. The majority (65.5%) of participants initiated prenatal care within the first trimester and were multiparous (69.8%). Half (50.1%) completed a high school education or less. Additional demographic details and comparisons across BMI class are given in Table 1.

**FIGURE 1 ∣:**
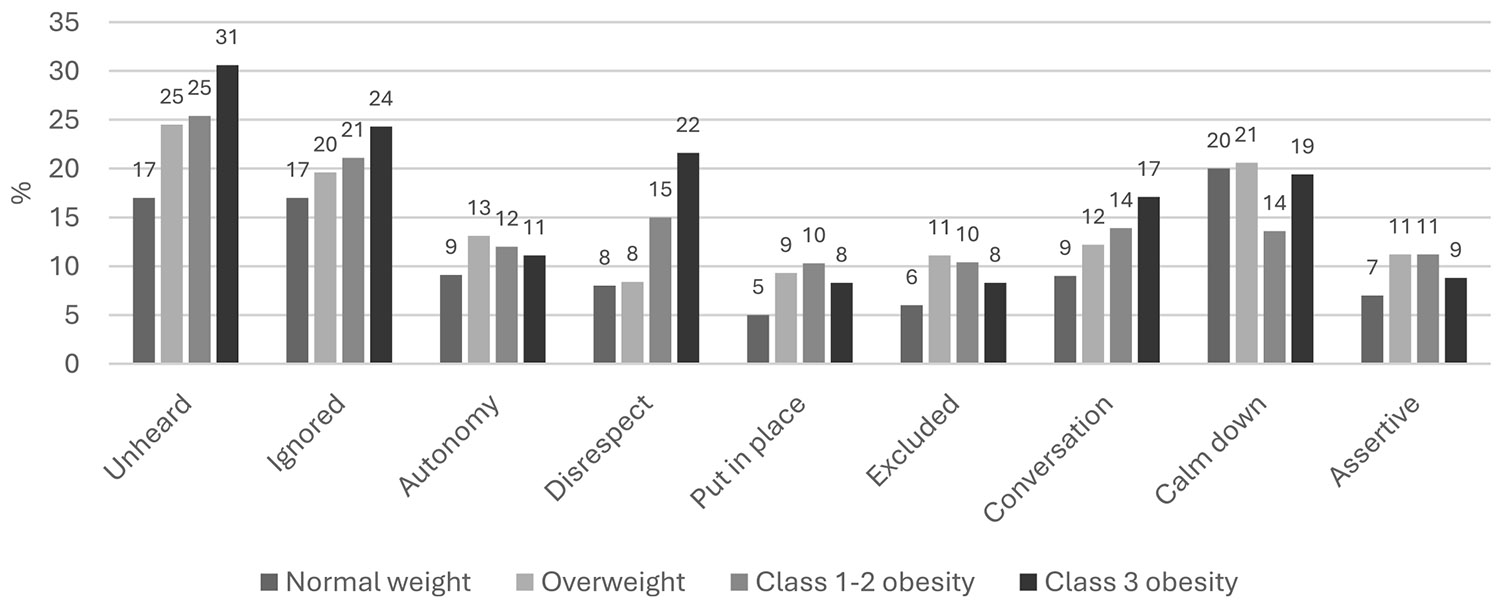
Percent of individuals who endorsed individual GRM Scale items, by early pregnancy BMI class, *n* = 368. Statements, from left to right: “I have felt unheard,” “My comments have been ignored,” “Someone challenged my autonomy,” “Someone has tried to ‘put me in my place’,” “I have been disrespected,” “I felt excluded from services or resources,” “Someone assumed I did not have much to contribute to the conversation regarding my care,” “Someone told me to calm down,” “Someone accused me of being angry when speaking assertively.” Bars indicate the percent of participants who endorsed ever experiencing each item during prenatal or childbirth care. BMI = body mass index, GRM = gendered racial microaggressions.

In total, 37.0% (*n* = 136) of participants reported experiencing at least one instance of GRM; by BMI class, rates were 33.0% (*n* = 33) of individuals with normal weight, 39.1% (*n* = 43) with overweight, 33.3% (*n* = 40) with class I–II obesity, and 52.6% (*n* = 20) with class III obesity (data not shown in table). Figure 1 presents the percentage of participants in each BMI class who endorsed individual GRM Scale items. The most frequently endorsed items, particularly among those with higher BMI, were “I have felt unheard” and “My comments have been ignored.”

The mean ± SD GRM Scale score reported among participants was 3.2 ± 7.2. Mean GRM score increased with increasing early pregnancy BMI; individuals with overweight, class I-II obesity, and class III obesity reported a mean ± SD GRM Scale score of 3.3 ± 7.4, 3.9 ± 8.2, and 4.8 ± 9.3, respectively, compared to a mean score of 1.7 ± 3.8 among individuals with normal weight (Table 2). In Tweedie regression models adjusted for maternal age, education, parity, and late prenatal care entry, the average GRM Scale score (95% confidence interval) was 2.0 (1.0–3.7) times higher among people with overweight, 2.1 (1.1–3.9) times higher among people with class I-II obesity, and 2.6 (1.2–5.6) times higher among people with class III obesity compared to normal weight. Additionally adjusting for race-ethnicity did not change the pattern of our results (Table S2). In analyses of effect measure modification by race-ethnicity, we found that associations between increasing BMI and GRM were strongest among Black and Hispanic individuals (interaction term *p*_heterogeneity_ = 0.011; Table 3). Associations among Asian individuals were similar in magnitude to associations in the overall study population but not statistically significant. All results were similar in sensitivity analyses imputing early pregnancy weight for participants missing a first trimester weight measure (Table S3).

## 4 ∣ Discussion

The findings from this work are unique in their description of discrimination related to race, ethnicity, and gender and associations with body size among a socioeconomically and culturally diverse, prospective cohort. Among 368 participants, roughly one-third reported experiencing GRM during pregnancy or childbirth care. We observed associations between higher early pregnancy BMI and increased frequency of GRM.

Our study builds on previous research examining interpersonal bias and discrimination in primary [16, 17] and perinatal care settings [16-18]. We add to emerging quantitative research showing BMI-based disparities in the provision of respectful perinatal care [19]. Weight bias—negative attitudes and stereotypes based on weight or body size [20] – can manifest as providers asking fewer questions, offering less information, building less rapport, and blaming, which contribute to care deficiencies [7, 18, 21]. In a survey of pregnant and postpartum women, roughly 20% of respondents reported experiencing weight stigma in a healthcare setting, describing feelings of guilt, shame, or judgment [17]. Similarly, racial stereotyping has been associated with healthcare communication gaps [16], which can negatively influence childbirth experiences such as feeling dismissed or ignored by providers [22]. Clinician implicit bias is associated with racial disparities in pain assessment and obstetric anesthesia use [23] and may contribute to the overuse of obstetric interventions [24]. Our finding that individuals with higher BMI more often felt unheard, ignored, and disrespected emphasizes intersections among weight-, race-, and gender-based discrimination.

Our finding that early pregnancy BMI may increase GRM has implications for research and policy on intersecting forms of discrimination in perinatal care. It underscores the need for medical and public health professionals to address how weight bias and stigma impact health equity, or the assurance of conditions that allow for the optimal health of all people [25]. There is increasing recognition that stereotypes of people with obesity (i.e., being unmotivated or unintelligent) often reflect racist and sexist policy and social structures [26-28]. Weight stigma may therefore overlap with and exacerbate interpersonal racial-ethnic or gendered discrimination, but weight bias remains under-addressed in intersectional perinatal health disparities research [28]. Investigators should include attitudes toward body size alongside other sources of marginalization [29] in health care encounters and examine characteristics to which individuals attribute discrimination to parse the influence of gender, race, body size, and their interactions. Implicit bias programming should include patients with diverse body sizes in designing and testing inclusive and respectful prenatal and childbirth interventions.

A strength of this work includes use of the validated GRM Scale to measure the construct of everyday racial and gender-based discrimination. The GRM Scale has shown good construct and criterion validity, with no evidence of differential item functioning to capture experiences of GRM among diverse Global Majority patients in perinatal care [9]. Our study also benefited from involvement of a CWG for GRM Scale adaptation and interpretation. However, our study is not without limitations. Howell et al. note that the sample used to adapt the scale lacked comprehensive representation of the Global Majority identities, which may limit generalizability [9]. We were unable to enroll individuals who did not speak either English or Spanish, which were the primary languages among our study hospital patient populations. Administering the scale during the birth hospitalization may have also influenced disclosure of healthcare-related GRM. We did not have data on provider type for prenatal or childbirth care. We lacked information on prepregnancy BMI and used early pregnancy weight as a proxy, which we cannot be certain captured the participants’ weight preceding their experiences of GRM during pregnancy or childbirth care. However, we confirmed the robustness of our results in a sensitivity analysis imputing early pregnancy weight for individuals whose first weight measure occurred after 14 weeks of gestation. Finally, we observed statistically significant heterogeneity in associations between BMI and GRM by race-ethnicity, but the precision of our effect measure modification analyses was limited by small sample size in racial and ethnic subgroups, and we were only able to present unadjusted effect estimates. Future studies should investigate this finding in large, diverse samples to further elucidate how body size and racial-ethnic identity may interact to shape perinatal experiences of interpersonal discrimination.

## 5 ∣ Conclusions

This study contributes to a growing body of research on discrimination in perinatal care from an intersectional perspective incorporating race, gender, and BMI. We found a positive association between early pregnancy BMI and frequency of GRM. Our results underscore how the multifaceted identities of pregnant and birthing individuals influence patient experience and suggest that addressing a potential “triple burden” of racism, sexism, and weightism may improve quality and equity in perinatal care.

## Supplementary Material

birt70051-sup-0001-supinfo

Additional supporting information can be found online in the Supporting Information section. Table S1: Adapted Gendered Racial Microaggressions Scale items. Table S2: Associations between early pregnancy BMI class and GRM Scale score, including adjustment for race-ethnicity, *n* = 368. Table S3: Sensitivity analysis for associations between early pregnancy BMI class and GRM Scale score, using imputed values of early pregnancy weight for individuals with late prenatal care entry, *n* = 368. Figure S1: Histogram of right-skewed, zero-inflated Gendered Racial Microaggressions Scale scores, *n* = 368. Mean = 3.2, standard deviation = 7.2, median = 0.0, interquartile range (Q1, Q3) = 0.0, 2.5, range (min, max) = 0.0, 45.0. Grms_ctns = continuous gendered racial microaggressions scale score. Figure S2: Directed Acyclic Graphs for the association between early pregnancy body mass index and gendered racial microaggressions. Abbreviations: BMI = body mass index, PNC = prenatal care, GWG = gestational weight gain.

## Figures and Tables

**TABLE 1 ∣:** Characteristics of VIBE study population by early pregnancy BMI classification, *n* = 368.

Characteristic, *n* (%) or mean (SD)	Total	Normal weight (*n* = 100)	Overweight (*n* = 110)	Class I–II obesity (*n* = 120)	Class III obesity (*n* = 38)	*p*
Total sample	368 (100)					
Age, mean ± SD	30.2 ± 5.9	29.0 ± 6.6	29.4 ± 5.8	31.5 ± 5.4	30.9 ± 5.7	0.007
Race-ethnicity, *n* (%)						0.001
Asian, non-Hispanic	39 (10.6)	19 (19.0)	12 (10.9)	8 (6.7)	0 (0.00)	
Black, non-Hispanic	139 (37.8)	25 (25.0)	40 (36.4)	50 (41.7)	24 (63.2)	
Hispanic/Latina	152 (41.3)	48 (48.0)	46 (41.8)	47 (39.2)	11 (29.0)	
Other, including multiple races, and prefer not to answer	38 (10.3)	8 (8.0)	12 (10.9)	15 (12.5)	3 (7.9)	
Education, *n* (%)^a^						0.09
High school or less	180 (50.1)	41 (43.2)	63 (57.8)	59 (50.4)	17 (44.7)	
Some college	87 (24.2)	19 (20.0)	25 (22.9)	32 (27.4)	11 (29.0)	
BA or higher	92 (25.6)	35 (36.8)	21 (19.3)	26 (22.2)	10 (26.3)	
Prenatal Care Initiation, *n* (%)						0.05
First trimester	241 (65.5)	76 (76.0)	64 (58.2)	78 (65.0)	23 (60.5)	
After first trimester	127 (34.5)	24 (24.0)	46 (41.8)	42 (35.0)	15 (39.5)	
Parity, *n* (%)						< 0.001
Nulliparous	111 (30.2)	47 (47.0)	36 (32.7)	23 (19.2)	5 (13.2)	
Multiparous	257 (69.8)	53 (53.0)	74 (67.3)	97 (80.8)	33 (86.8)	
Hypertensive Disorders of Pregnancy, *n* (%)						< 0.001
Yes	114 (31.0)	19 (19.0)	32 (29.1)	43 (35.8)	20 (52.6)	
No	254 (69.0)	81 (81.0)	78 (70.9)	77 (64.2)	18 (47.4)	
Preterm birth (< 37 weeks), *n* (%)						0.34
Yes	35 (9.5)	5 (5.0)	13 (11.8)	13 (10.8)	4 (10.5)	
No	333 (90.5)	95 (95.0)	97 (88.2)	107 (89.2)	34 (89.5)	
Gestational age at birth, mean ± SD	38.9 ± 1.7	39.3 ± 1.4	38.6 ± 2.1	39.0 ± 1.5	38.6 ± 1.8	0.02
Gestational weight gain (*z*-score)^a^						< 0.001
< −1	156 (43.0)	12 (12.5)	59 (54.1)	71 (59.2)	14 (36.8)	
−1 to 1	172 (47.4)	53 (55.2)	47 (43.1)	48 (40.0)	24 (63.2)	
> 1	35 (9.6)	31 (32.3)	3 (2.8)	1 (0.8)	0 (0.0)	

*Note:* In total, *n* = 9 (2.4%) observations missing for education and 2 (0.1%) for gestational age at birth.

Abbreviations: BMI = body mass index, SD = standard deviation.

a Gestational weight gain measured as a *z*-score based on previously published BMI class-specific weight-gain-for-gestational-age charts, as in: Hutcheon et al. [15].

**TABLE 2 ∣:** Associations between early pregnancy BMI class and GRM Scale score, *n* = 368.

BMI class^a^	Mean ± SD score	Unadjusted *β*^a^ (95% CI)	Adjusted *β*^b^(95% CI)
Normal weight (*n* = 100)	1.7 ± 3.8	Ref	Ref
Overweight (*n* = 110)	3.3 ± 7.4	2.0 (1.1–3.6)	2.0 (1.0–3.7)
Class I–II obesity (*n* = 120)	3.9 ± 8.2	2.3 (1.3–4.1)	2.1 (1.1–3.9)
Class III obesity (*n* = 38)	4.8 ± 9.3	2.9 (1.4–6.0)	2.6 (1.2–5.6)

*Note:* Mean ± SD GRM Scale scores are based on the observed data within each BMI category. Unadjusted and adjusted *β* coefficients are derived from Tweedie regression models, which accommodates zero-inflated and right-skewed data, and represent model-based mean differences in GRM Scale score by BMI category. *β* coefficients are interpreted as the multiplicative effect of BMI class on the expected value of GRM Scale score.

Abbreviations: BMI = body mass index, CI = confidence interval, GRM = gendered racial microaggressions, SD = standard deviation.

a Normal weight: 18.5 ≤ BMI < 25, overweight: 25 ≤ BMI < 30, class 1–2 obesity: 30 ≤ BMI < 40, class 3 obesity: BMI ≥ 40.

b Adjusted for age, education, parity, and late prenatal care entry.

**TABLE 3 ∣:** Effect measure modification of the association between early pregnancy BMI class and GRM Scale score by race-ethnicity, *n* = 368.

BMI class^a^	Mean ± SD score	Unadjusted *β*^a^ (95% CI) *p*_heterogeneity_ = 0.011
Asian (*n* = 39)		
Normal weight (*n* = 19)	1.2 ± 2.1	Ref
Overweight (*n* = 12)	1.8 ± 3.6	1.6 (0.4–6.6)
Obesity (*n* = 8)	2.5 ± 4.6	2.2 (0.5–9.9)
Black (*n* = 139)		
Normal weight (*n* = 25)	1.1 ± 3.0	Ref
Overweight (*n* = 40)	3.1 ± 7.6	2.8 (0.8–9.6)
Obesity (*n* = 74)	3.5 ± 7.5	3.1 (1.0–9.9)
Hispanic, including White Hispanic (*n* = 152)		
Normal weight (*n* = 48)	1.6 ± 3.2	Ref
Overweight (*n* = 46)	4.4 ± 8.6	2.8 (1.2–6.4)
Obesity (*n* = 58)	3.5 ± 7.0	2.3 (1.0–5.1)
Other, including multiple races, and NR/Prefer not to answer (*n* = 38)		
Normal weight (*n* = 8)	5.4 ± 8.8	Ref
Overweight (*n* = 12)	1.3 ± 3.0	0.2 (0.0–1.5)
Obesity (*n* = 18)	9.1 ± 14.7	1.7 (0.4–6.4)

*Note:* Mean ± SD GRM Scale scores are based on the observed data within each BMI category. Unadjusted *β* coefficients are derived from Tweedie regression models, which accommodates zero-inflated and right-skewed data, and represent model-based mean differences in GRM Scale score by BMI category. *β* coefficients are interpreted as the multiplicative effect of BMI class on the expected value of GRM Scale score.

Abbreviations: BMI = body mass index, CI = confidence interval, GRM = gendered racial microaggressions, SD = standard deviation.

a Normal weight: 18.5 ≤ BMI < 25, overweight: 25 ≤ BMI < 30, class 1–2 obesity: 30 ≤ BMI < 40, class 3 obesity: BMI ≥ 40.

## Data Availability

The data that support the findings of this study are available on request from the corresponding author. The data are not publicly available due to privacy or ethical restrictions.
