# Supplementary material for: Early Pregnancy Body Mass Index and Experiences of Gendered Racial Microaggressions in a Multiracial, Multiethnic Prospective Cohort
Source: Birth. Author manuscript; Available in PMC 2026 Jun 15. (PMC13267137; doi:10.1111/birt.70051)
Supplement: birt70051-sup-0001-supinfo [file NIHMS2176462-supplement-birt70051-sup-0001-supinfo.pdf]

Early Pregnancy Body Mass Index and Experiences of Gendered Racial Microaggressions in a Multiracial, Multiethnic Prospective Cohort

Kimberly B. Glazer PhD MPH, Natalie Boychuk MPH, Frances M. Howell MA, Micki Burdick MA PHD, Sarah Nowlin, PhD MSN RN, Sheela Maru, MD MPH, Oluwadamilola Oshewa, Maria Monterroso, Erynne Jackson MPH, Katharine McCarthy PhD MPH, Alva Rodriguez, Jennifer Lewey MD MPH, Elizabeth A. Howell MD MPP, Lisa Levine MD MSCE, Teresa Janevic PhD MPH

Affiliations: University of Pennsylvania Perelman School of Medicine, Department of Obstetrics and Gynecology (Dr. Glazer, Ms. Oshewa, Ms. Monterroso, Dr. Howell, Dr. Levine); University of Pennsylvania Perelman School of Medicine, Department of Biostatistics, Epidemiology, and Informatics (Dr. Glazer); Icahn School of Medicine at Mount Sinai, Department of Population Health Science and Policy (Dr. Glazer, Dr. Nowlin, Dr. McCarthy); Icahn School of Medicine at Mount Sinai, Department of Obstetrics, Gynecology and Reproductive Science (Dr. Glazer, Dr. Maru, Dr. McCarthy); Columbia University Mailman School of Public Health, Department of Epidemiology (Dr. Janevic, Ms. Boychuk, Ms. Howell, Ms. Jackson); University of Delaware, Department of Women and Gender Studies (Dr. Burdick); Mount Sinai Health System, Center for Nursing Research and Innovation (Dr. Nowlin); Icahn School of Medicine at Mount Sinai, Department of Global Health and Health Systems Design (Dr. Maru, Ms. Rodriguez); NYC Health + Hospitals, Elmhurst Department of Obstetrics & Gynecology (Dr. Maru); University of Pennsylvania Perelman School of Medicine, Division of Cardiovascular Medicine (Dr. Lewey)

Table of Contents

Table S1. Adapted Gendered Racial Microaggressions Scale items

Figure S1. Histogram of right-skewed, zero-inflated GRM Scale scores (n=368)

Figure S2. Directed acyclic graph

Table S2. Associations between early pregnancy BMI class and GRM Scale score, including adjustment for race-ethnicity, n=368

Table S3. Sensitivity analysis for associations between early pregnancy BMI class and GRM Scale score, using imputed values of early pregnancy weight for individuals with late prenatal care entry, n=368

Table S1. Adapted Gendered Racial Microaggressions Scale items

Original GRMS Abbreviated Survey Item	Adapted GRMS Survey Item
**I have felt unheard**	I have felt unheard
**My comments have been ignored**	My comments have been ignored
**Someone challenged my authority**	Someone challenged my **autonomy** [a]
**I have been disrespected in the workplace**	I have been disrespected
**Someone has tried to "put me in my place"**	Someone has tried to "put me in my place"
**Felt excluded from networking opportunities**	Felt excluded from **services or resources** [a]
**Assumed I did not have much to contribute to the conversation**	Assumed I did not have much to contribute to the conversation **regarding my care** [a]
**Someone told me to calm down**	Someone told me to calm down
**Someone accused me of being angry when speaking calm**	Someone accused me of being angry when speaking **assertively** [a]

a. Reflects survey items that have been adapted to the obstetric context

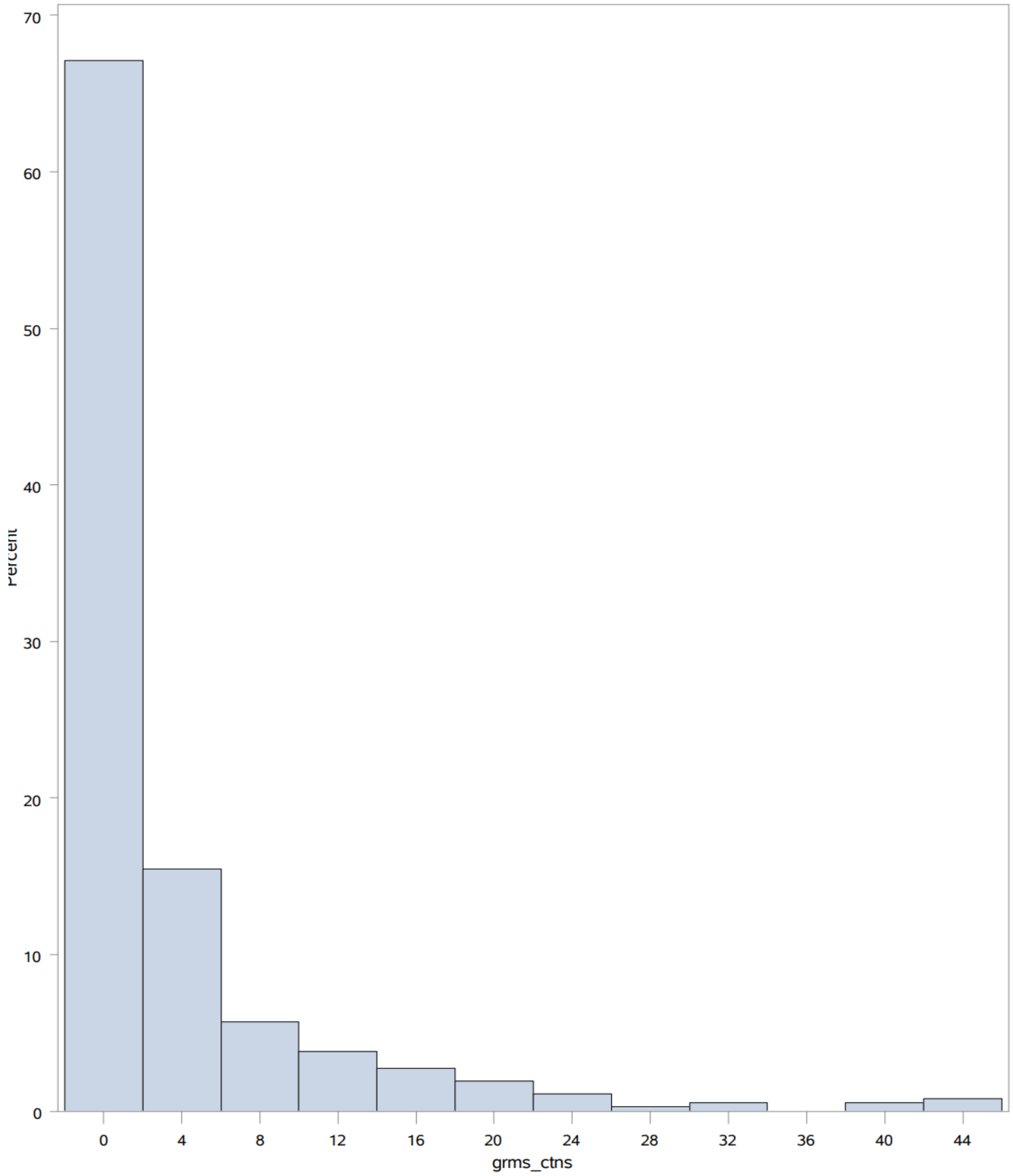

Figure S1. Histogram of right-skewed, zero-inflated Gendered Racial Microaggressions Scale scores, n=368. Mean=3.2, standard deviation=7.2, median=0.0, interquartile range (Q1, Q3)=0.0, 2.5, range (min, max)=0.0, 45.0. Grms_ctns=continuous Gendered Racial Microaggressions Scale Score.

Figure S2. Directed Acyclic Graphs for the association between early pregnancy body mass index and gendered racial microaggressions. Abbreviations: BMI=body mass index, PNC=prenatal care, GWG=gestational weight gain, GRM=gendered racial microaggressions.
Minimal sufficient covariate set to control for confounding of the association between early pregnancy BMI and GRM includes education, age, parity, and late prenatal care entry. GWG not included as adjustment variable given position downstream of early pregnancy BMI on the hypothesized causal pathway. Race-ethnicity is hypothesized to be an effect modifier.

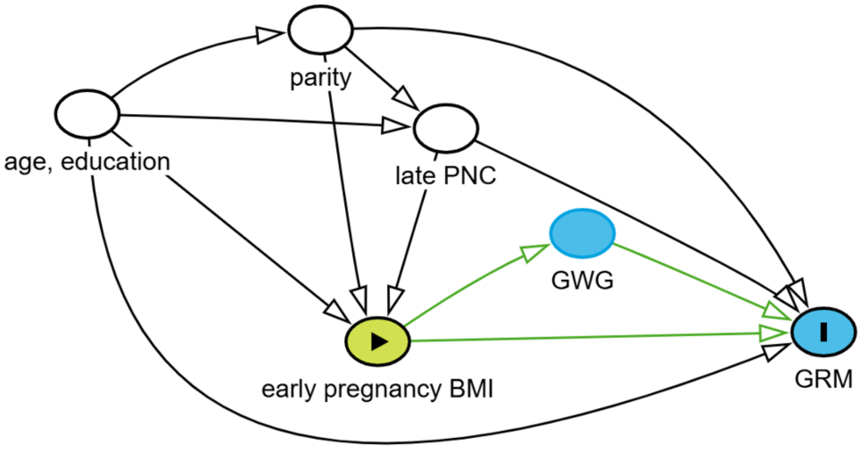

Table S2. Associations between early pregnancy BMI class and GRM Scale score, including adjustment for race-ethnicity, n=368

**BMI class**[a]	**Mean ± SD score**	**Unadjusted β (95% CI)**	**Adjusted β**[b] **(95% CI)**[c]
Normal weight (n=100)	1.7 ± 3.8	Ref	Ref
Overweight (n=110)	3.3 ± 7.4	2.0 (1.1-3.6)	2.2 (1.2-4.0)
Class I-II obesity (n=120)	3.9 ± 8.2	2.3 (1.3-4.1)	2.2 (1.1-4.2)
Class III obesity (n=38)	4.8 ± 9.3	2.9 (1.4-6.0)	2.9 (1.3-6.2)

[a]Normal weight: 18.5≤BMI<25, overweight: 25≤BMI<30, class 1-2 obesity: 30≤BMI<40, class 3 obesity: BMI≥40; [b]Adjusted for maternal age, education, parity, late prenatal care entry, and race-ethnicity (Black; Hispanic, including White Hispanic, or Asian; Other, including multiple races, and not reported/prefer not to answer).

Mean±SD GRM Scale scores are based on the observed data within each BMI category. Unadjusted and adjusted β coefficients are derived from Tweedie regression models, which accommodates zero-inflated and right-skewed data, and represent model-based mean differences in GRM score by BMI category. β coefficients are interpreted as the multiplicative effect of BMI class on the expected value of GRM score.

Abbreviations: BMI=body mass index; CI=confidence interval; GRM=gendered racial microaggressions; SD=standard deviation.

Table S3. Sensitivity analysis for associations between early pregnancy BMI class and GRM Scale score, using imputed values of early pregnancy weight for individuals with late prenatal care entry, n=368

**BMI class**[a]	**Unadjusted β (95% CI)**	**Adjusted β**[b] **(95% CI)**[c]
Normal weight	Ref	Ref
Overweight	1.9 (0.9-3.7)	1.8 (0.9-3.8)
Class I-II obesity	1.8 (1.0-3.5)	1.7 (0.9-3.4)
Class III obesity	2.5 (1.1-5.4)	2.3 (1.0-5.1)

[a]Normal weight: 18.5≤BMI<25, overweight: 25≤BMI<30, class 1-2 obesity: 30≤BMI<40, class 3 obesity: BMI≥40;
[b]Adjusted for maternal age, education, parity, and late prenatal care entry.
Mean(SD) GRM scores are based on the observed data within each BMI category. Unadjusted and adjusted β coefficients are derived from Tweedie regression models, which accommodates zero-inflated and right-skewed data, and represent model-based mean differences in GRM score by BMI category. β coefficients are interpreted as the multiplicative effect of BMI class on the expected value of GRM score.
Abbreviations: BMI=body mass index; CI=confidence interval; GRM=gendered racial microaggressions; SD=standard deviation.
